# Critical care in a maternity hospital: a retrospective observational study

**DOI:** 10.1186/cc14610

**Published:** 2015-03-16

**Authors:** F Kavanagh, I Browne

**Affiliations:** 1 National Maternity Hospital, Dublin, Ireland

## Introduction

Pregnancy and labour usually progress uneventfully; however, serious complications can occur and develop rapidly, necessitating critical care admission and support. Successive confidential enquires have highlighted deficiencies in this area and suboptimal care leading to increased morbidity and mortality [1]. The National Maternity Hospital is the largest maternity hospital in the Republic of Ireland where a total of 8,954 babies were delivered in 2013. It is a stand-alone institution and onsite facilities include a two-bed dedicated anaesthesia lead high-dependency unit.

## Methods

A retrospective observational study was carried out from January 2011 to January 2014 especially looking at the following parameters: admitting diagnosis, demographics, length of stay and the number of admissions requiring transfer for tertiary-level care.

## Results

In total, 29,344 deliveries occurred. A total of 376 HDU admissions were recorded in this period, representing 1.28% of all admissions. The average age of patients admitted to the HDU was 34 years. The predominant reasons for admission were hypertensive disease of pregnancy (49.7%), haemorrhage (antepartum/postpartum) (36.4%), sepsis (4.2%) and other reasons (11.1%) including cardiac rhythm disturbances, neurological complications and pre-existing medical disease. In 2013 the average length of stay was 2 days. A total 6.1% of those admitted to HDU required transfer for tertiary-level ICU care in other centres during the study period, this represented 0.07% of all deliveries.

## Conclusion

There is significant demand within our institution for HDU care for our patients, with the number of admissions increasing in 2013. The main admitting diagnoses are hypertensive disease of pregnancy and haemorrhage with an increase in the number of patients being admitted for management of sepsis in 2013. This highlights the increasing awareness, recognition and management of this condition in pregnancy. The increased number of HDU admissions in 2013 could also be explained by the recent Introduction of an early warning score for the deteriorating patient in our hospital but this would require further evaluation. The low number of transfers of patients to other tertiary centres underpins the importance of an anaesthesia lead service.
